# Pulmonary artery stenosis in Takayasu disease mimicking pulmonary embolism on perfusion lung scan: A case report

**DOI:** 10.3389/fnume.2023.1122046

**Published:** 2023-02-10

**Authors:** Chaimae Sebbar, Soufiane Hiroual, Nathalie Kouassi, Mohamed Aziz Bsiss, Aboubaker Matrane

**Affiliations:** ^1^ Mohamed VI University Hospital, Nuclear Medicine Department, Cadi Ayyad University, Marrakesh, Morocco; ^2^ TTA University Hospital, Nuclear Medicine Department, Abdelmalek Esaadi University, Tangier, Morocco

**Keywords:** perfusion lung scan, takayasu arteritis, pulmonary embolism, pulmonary artery stenosis, unilateral absence of perfusion

## Abstract

Lung scan is an accurate and noninvasive tool for evaluating the distribution of lung perfusion. We present a rare case of total absence of perfusion on lung scan to the right lung with two contralateral defects due to severe occlusion of the right pulmonary artery, as well as segmental arteries in the left lung in a young female woman diagnosed with Takayasu Arteritis. We highlight the similarities of the findings between pulmonary arteritis in Takayasu disease and thromboembolic disease and the importance of careful interpretation of perfusion lung scan in order to avoid misdiagnosis that could endanger the patient's life.

## Introduction

Absence of perfusion to an entire lung on perfusion lung scan with a preserved ventilation is an uncommon yet dramatic finding, accounting for 2% of all lung scans (1). Only a minority of these are due to pulmonary embolism (1). Therefore, other possibilities should be strongly considered to avoid a false positive diagnosis. The most common causes of this finding are external compression by a bronchogenic carcinoma and congenital heart disease (2). Occlusion of a pulmonary artery due to arteritis may result in this appearance but is an uncommon cause (2). We present a patient with Takayasu arteritis (TA) subjected to perfusion lung scan who showed a unilateral absence of perfusion to the right lung as well as contralateral perfusion defects, mimicking pulmonary embolism (PE).

## Case presentation

**Figure 1:**
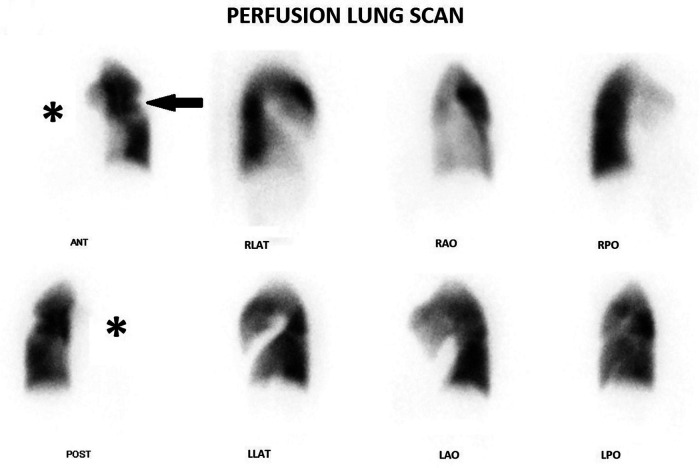
Planar images show a total absence of perfusion to the right lung (star) with a contralateral defect (arrow).

A 36 years old female patient, diagnosed with Takayasu Arteritis one year ago based on clinical, biological and imaging features. She was referred to our department for a perfusion lung scan for suspicion of pulmonary embolism after presenting with chest pain and acute exacerbation of her chronic dyspnea. The patient underwent perfusion lung scan after injection of 148 MBq (4 mCi) of 99mTc-MAA in the supine position. Six views were obtained with a gamma camera with a low energy high resolution parallel hole collimator. Pulmonary ventilation scintigraphy was not available in our department. We completed with a single photon emission tomography combined with computed tomography (SPECT/CT). Planar images (Figure 1) showed a total absence of perfusion to the right lung with a contralateral perfusion defect.

**Figure 2:**
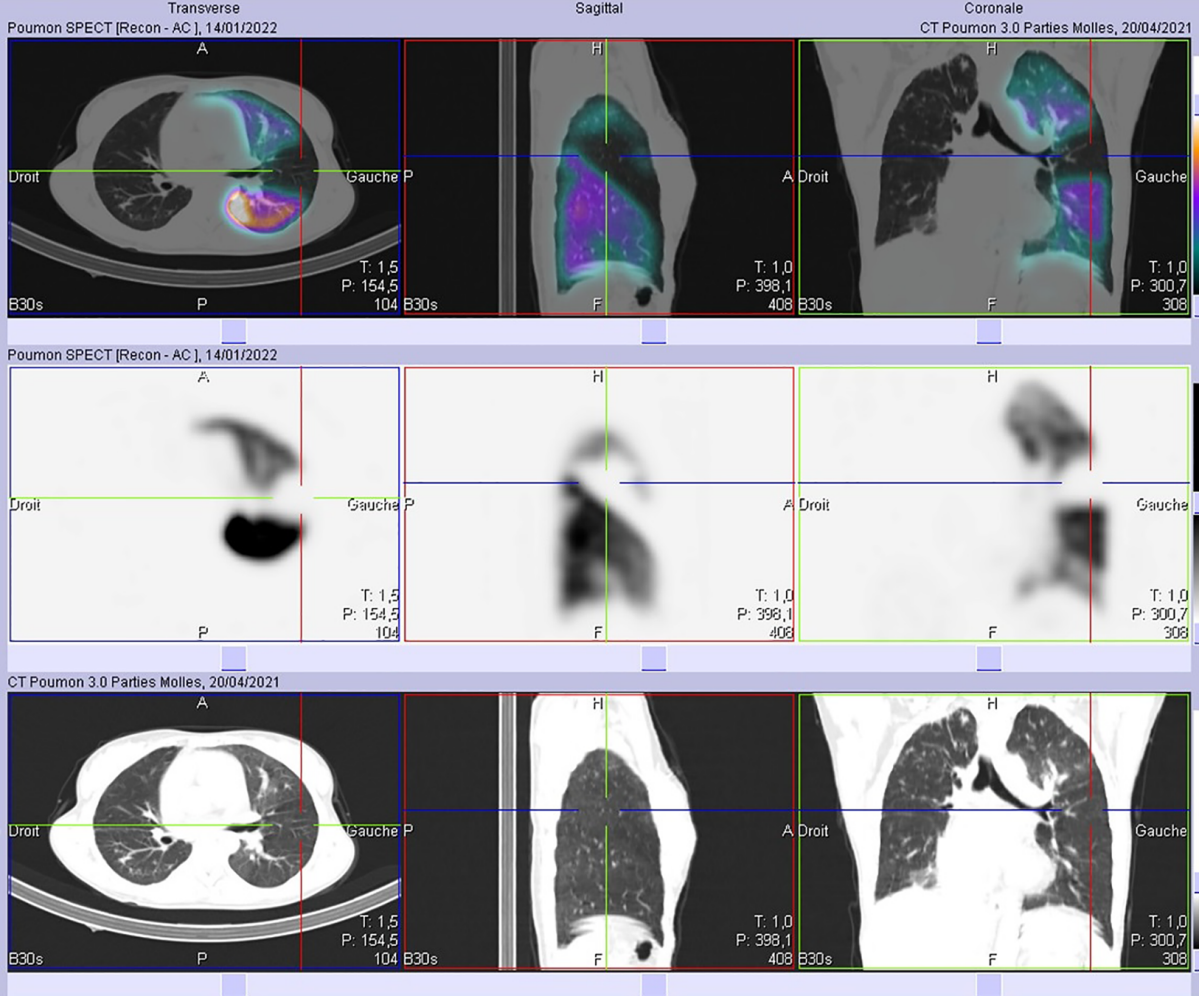
No abnormal parenchymal findings were seen in both lung fields adjacent to the defects on SPECT-CT images.

**Figure 3:**
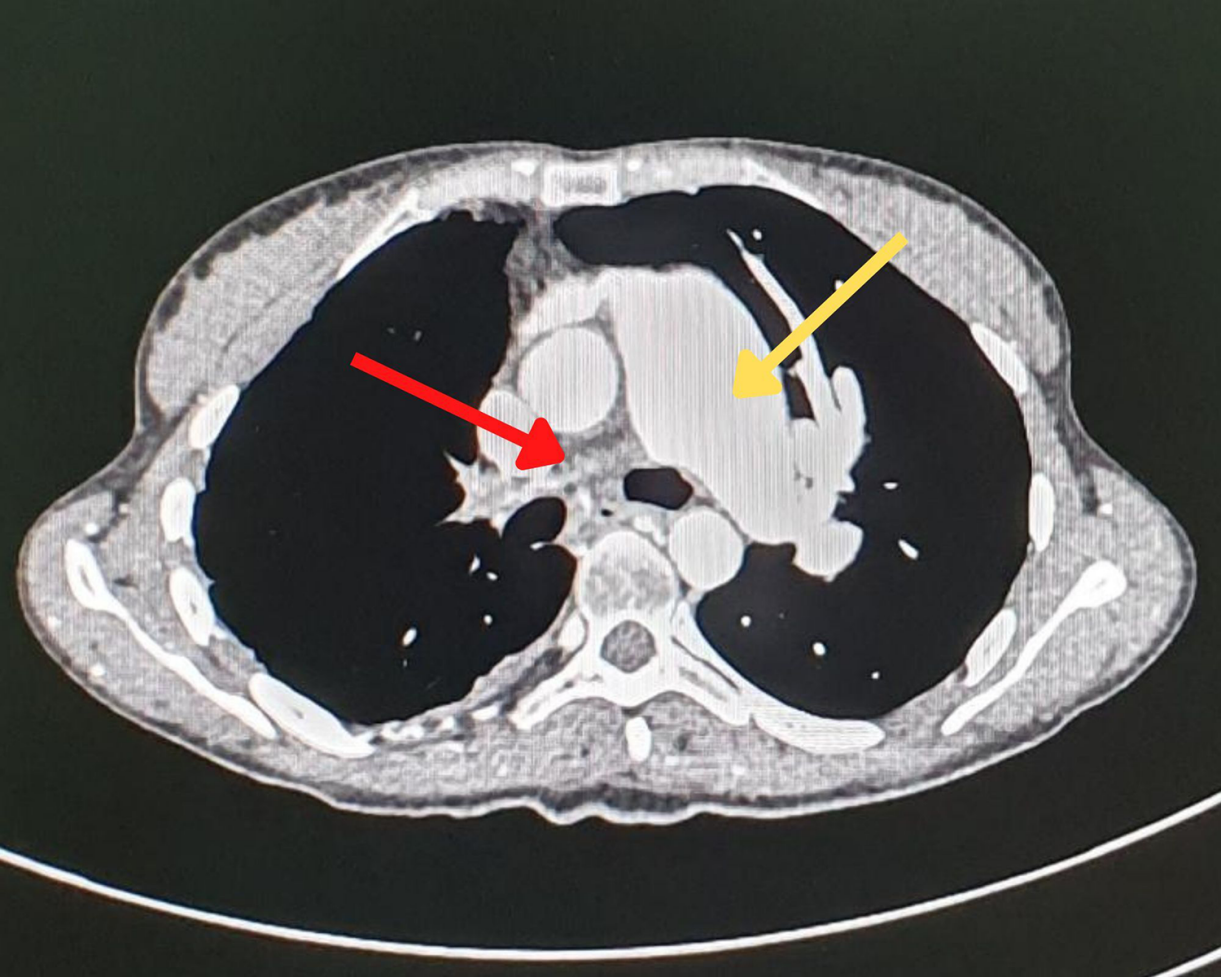
Chest angiographic CT showing a dilatation of the main pulmonary artery (yellow arrow) and a lack of the opacification of the right pulmonary artery (red arrow), consistent with a severe stenosis of the latter.

Subsequent SPECT/CT (Figure 2) revealed segmental defects in the lingula and the anterior and posterior segments of the culmen with no lung parenchymal changes in the corresponding CT. We suspected severe occlusion of pulmonary arteries as well as pulmonary embolism. Because a total absence of perfusion to an entire lung is hardly due to pulmonary embolism, we recommended that the patient undergoes a chest angiographic CT (Figure 3) which confirmed a severe stenosis of the right pulmonary artery and the left upper lobar artery as well as inferior segmental ones. This finding in our patient was consistent with a severe involvement of pulmonary arteries in Takayasu disease rather than pulmonary embolism. The patient was initially on prednisolone 5 mg per day, treatment with high-dose prednisolone (120 mg per day) and azathioprine was started right away and symptoms gradually improved within one month. The patient was then lost to further follow up, all attempts to locate her *via* phone were unsuccessful.

## Discussion

Lung scan is an accurate and noninvasive tool for evaluating the distribution of lung perfusion. The main indication is evaluation of suspected pulmonary embolism with a fairly high accuracy especially with the introduction of single photon emission tomography (SPECT) combined with low dose CT (3). However, the rate of false positive scans is not negligible (4).

Unilateral absence of perfusion to an entire lung on a radionuclide scan is a very rare occurrence. According to a series of 607 lung scans, it accounts for 2% of cases (1). This appearance classically suggests an external compression of the pulmonary arteries by a tumor, mostly bronchogenic carcinoma. Less common entities include congenital heart disease, pneumonectomy and pulmonary embolism (2). The latter being very unlikely particularly with a normal contralateral lung perfusion (5).

Takayasu arteritis is a chronic vasculitis of unknown origin that involves the aorta and its main branches and leads to vascular thickening, stenosis and occlusion (6). Besides the aorta and its branches, pulmonary arteries can also be involved. Their involvement in TA is of late diagnosis because of non-specific symptoms, but it's not infrequent even in the absence of symptoms. Its prevalence varies widely among series and it could be as high as 80% (7).

Radionuclide lung scanning is a simple and safe way to evaluate pulmonary arteries involvement in Takayasu disease (8). The abnormalities in lung scan in Takayasu arteritis can be quite similar to those in pulmonary embolism, showing decreased or absent perfusion corresponding to specific blood vessels. Despite the difficulty to differentiate Takayasu arteritis from pulmonary embolism without clinical findings, there are some differences in the lung scans between these two diseases. In pulmonary embolism, perfusion defects often resolve, particularly in younger patients. On the other hand, perfusion defects in Takayasu arteritis do not change for a long time. These findings might be useful in ruling out pulmonary embolism (8). In contrast, it is generally uncomplicated to differentiate between stenotic lesions and emboli on an angiographic CT. stenotic lesions may appear as areas of narrowing or obstruction within the blood vessel, while emboli may appear as denser, round or oval-shaped areas within the vessel.

Classically, pulmonary arteritis in TA results in multiple perfusion mismatch defects on Ventilation/perfusion lung scan. Although TA can cause unilateral absence of perfusion, in rare cases it can mimic acute pulmonary embolism. As far as we are aware, our case is the third report of unilateral absence of perfusion occurring in a Takayasu arteritis patient. Two prior reports have been described where the diagnosis of TA was not yet known at the time of lung scan which made the interpretation quite tricky and led to misdiagnosis as pulmonary embolism (PE) (9, 10).

In our case, although the diagnosis of TA was established before perfusion lung scan, PE was included in the differential diagnosis because of the clinical presentation, the contralateral systematized defects and the high risk of association of thromboembolism with vasculitis (11). This finding in our patient suggests a long evolution of the disease and thus TA is at the chronic stage, as it was demonstrated in a Japanese study (12).

## Conclusions

Our case highlights two important points: (I) a rare occurrence of total absence of perfusion to an entire lung due to an uncommon etiology which is TA. (II) interpretation of perfusion lung scan should be careful and clinical features as well as medical history should be taken into account before giving a diagnosis that could endanger the life of the patient.

## Data Availability

The original contributions presented in the study are included in the article/Supplementary Material, further inquiries can be directed to the corresponding author.
